# Determination of Lipoprotein Z-Specific IgA in Tuberculosis and Latent Tuberculosis Infection

**DOI:** 10.3389/fcimb.2017.00495

**Published:** 2017-11-30

**Authors:** Jia-ni Xiao, Yanqing Xiong, Yingying Chen, Yang-jiong Xiao, Ping Ji, Yong Li, Shu-jun Wang, Guo-ping Zhao, Qi-jian Cheng, Shui-hua Lu, Ying Wang

**Affiliations:** ^1^Department of Microbiology and Immunology, Shanghai Institute of Immunology, Shanghai Jiao Tong University School of Medicine, Shanghai, China; ^2^Key Laboratory of Medical Molecular Virology of MOE/MOH, Shanghai Public Health Clinical Center, Fudan University, Shanghai, China; ^3^Shanghai-MOST Key Laboratory of Health and Disease Genomics, Chinese National Human Genome Center at Shanghai, Shanghai, China; ^4^Department of Respiratory Diseases, Ruijin Hospital, Shanghai Jiao Tong University School of Medicine, Shanghai, China

**Keywords:** lipoprotein Z, IgA, tuberculosis, latent tuberculosis infection, screening

## Abstract

Tuberculosis (TB) remains one of the most severe infectious diseases. It is still of paramount importance to establish more accurate, rapid, and efficient diagnostic methods. Since infection with *Mycobacterium tuberculosis* (*M. tb*) is largely mediated through the respiratory tract, IgA responses against mycobacterial proteins are worthy of investigation for their potential clinical utility. In this study, the IgA response targeting lipoprotein Z (LppZ) was determined by using a homemade ELISA with plasma of TB patients (*N* = 125), LTBI individuals (*N* = 92), healthy controls (HCs) (*N* = 165), as well as TB patients undergoing anti-TB treatment (*N* = 9). In parallel the antigen-specific IFN-γ release from PBMCs triggered by LppZ and *M. tb*-specific ESAT-6 or CFP-10 was detected by using an ELISPOT assay. It was found that the LppZ-specific IgA level was dramatically higher in TB patients than in HCs (*p* < 0.0001). Compared to that before anti-TB treatment, the LppZ-specific IgA level decreased substantially after 2 months of anti-TB treatment (*p* = 0.0297) and remained at low levels until the end of the treatment. What is more, pulmonary TB patients exhibited significantly higher LppZ-specific IgA-values than extra-pulmonary TB patients (*p* = 0.0296). Interestingly, the LppZ-specific IgA-values were negatively correlated to the amounts of IFN-γ released in response to LppZ with statistical significance (*r* = −0.5806, *p* = 0.0002). LppZ-specific IgA level was also higher in LTBI individuals than in HCs (*p* < 0.0001). Additionally there were some PPD^+^ HC individuals with high LppZ-specific IgA levels but the potential of this assay for identifying leaky LTBI in PPD^+^ HCs needs to be further investigated through follow-up studies. The sensitivity of detecting TB solely with ESAT-6 or CFP-10-specific IFN-γ release was increased by including the LppZ-specific IgA results, respectively, from 86.11 to 100% and 88.89 to 100%; the sensitivity of screening for LTBI was increased from 80.36 to 83.93% and 57.14 to 69.64%, respectively. The higher LppZ-specific IgA responses in TB and LTBI populations than in controls indicated high immunoreactivity to LppZ upon *M. tb* infection. Although the assay was not efficient enough for independent application in sero-diagnosis, LppZ-specific IgA might become a complementary biomarker for the improvement of TB and LTBI screening.

## Introduction

Tuberculosis (TB), which is caused by *Mycobacterium tuberculosis* (*M. tb*) infection, remains one of the biggest infectious disease threats worldwide. One-third of the global population has been infected (Dye et al., 1999). According to the Global Tuberculosis Report 2016 issued by World Health Organization (WHO), there were 10.4 million newly onset TB patients and 1.4 million were killed by TB in 2015. China is among the countries with high TB burden, as demonstrated by the incidence of 918,000 cases and the mortality of 37,600 cases in 2015, ranking third following India and Indonesia (WHO, 2016). Improved TB prevention and control is urgently needed, worldwide and in China, and approaches to optimize current diagnostic methods are of great significance and value.

Currently conventional TB diagnosis mainly relies on clinical symptoms, chest X-rays, and pathogenic microorganism confirmation through sputum microscopy and sputum culture (Baumann et al., 2014). Although identification of mycobacterium bacilli in sputum samples by culture is the gold standard, the long time required together with practical difficulties in bacilli culture limit its successful application in the clinic. The tuberculin skin test (TST) is strongly recommended for TB diagnosis in populations that have not been vaccinated with Bacillus Calmette-Guérin (BCG) (Horsburgh, 2004; Pai et al., 2014). The test is based on a delayed-type hypersensitivity response to purified protein derivative (PPD) containing at least 200 shared antigens of BCG and *M. tb* (Lalvani and Pareek, 2010). Since BCG vaccination is obligatory in China, the TST is not satisfactory either for detecting active TB or for discriminating between *M. tb*-infected and uninfected people in a healthy population (Araujo et al., 2008; Zhang et al., 2010). To increase the specificity in TB diagnosis, an *M. tb*-specific cellular response-based method, known as an interferon gamma release assay (IGRA) has been developed. This detects IFN-γ releasing cells when they are stimulated with two specific antigens, early secretary antigen target-6 (ESAT-6) and culture filtrate protein-10 (CFP-10) (Berthet et al., 1998; Guinn et al., 2004), which are absent from BCG strains and most non-tuberculosis mycobacteria (NTM) (Andersen et al., 2000). Two commercial IGRA kits, QuantiFERON-TB Gold In-Tube (QFT) and T-SPOT.TB, have been applied in TB diagnosis as well as latent tuberculosis infection (LTBI) screening (Ewer et al., 2006; Guio et al., 2011). However, variable sensitivity in detecting active TB in healthy populations and low specificity in distinguishing active TB from LTBI make it necessary to develop alternative diagnostic methods.

Sero-diagnosis is applicable in many infectious diseases such as hepatitis, AIDS etc. However, no successful sero-diagnosis methods have been commercialized for TB based on *M. tb* antigen-specific IgG responses (Baumann et al., 2014; Chen et al., 2015). Owing to the fact that *M. tb* infection is mainly mediated through the respiratory tract, mucosal immunity-derived *M. tb*-specific IgAs have been demonstrated to play important roles in the defense against *M. tb* (Kerr, 1990; Casadevall, 2017). IgA-deficient mice were found to be more susceptible to *Mycobacterium bovis* infection and had higher bacterial loads in the lungs and bronchoalveolar lavage as well as lower IFN-γ and TNF-α production in the lungs when compared to wild-type mice (Rodríguez et al., 2005). One recent study revealed that *M. tb-*specific IgA antibody could inhibit mycobacterial infection in an epithelial cell line while IgG antibody promoted *M. tb* infection (Zimmermann et al., 2016). Moreover, purified secretory IgA from human colostrum mediated protection against *M. tb* infection in mice (Alvarez et al., 2013). In addition to the demonstration of protective roles, elevated IgA responses targeting *M. tb* antigens have been evaluated for their potential in diagnosis. For instance, Bezerra et al. (2009) reported that compared to healthy controls (HCs), pulmonary TB (PTB) patients displayed specific IgA response to glycolipid antigen with an assay sensitivity of 88% and the specificity of 89%. Legesse et al. (2013) described the presence of increased levels of IgA against ESAT-6/CFP-10 and Rv2031 in the sera of culture-confirmed PTB patients when compared to healthy *M. tb-*infected and non-infected individuals. IgA targeting other *M. tb* antigens, such as LAM, PE35 (Baumann et al., 2014), P-90 (Conde et al., 2004), PstS3, MPT83 (Baumann et al., 2015) was also detectable in *M. tb-*infected individuals.

Mycobacterial lipoproteins have been extensively studied due to their “double-edged sword” features, contributing to both virulence and immunity during *M. tb* infection (Becker and Sander, 2016). Mycobacterial lipoproteins function in nutrient uptake, drug export, cell wall homoeostasis, and host–pathogen interaction (Rezwan et al., 2007; Buddelmeijer, 2015). Lipoprotein Z (LppZ), encoded by *rv3006*, is a conserved lipoprotein classified in the category of “enzymes and metabolic activities” (Sutcliffe and Harrington, 2004). It has been reported as a novel B cell antigen (Sartain et al., 2006) and one of the most immunogenic proteins with high antibody-to-protein ratio among culture filtrate proteins (Målen et al., 2008). Our previous study revealed that LppZ exhibited strong cellular immunoreactivity in active TB (Xiao et al., 2016). In this study, we determined IgA immune responses to LppZ among three human cohorts: TB patients, LTBI individuals and HCs. Antigen-specific IFN-γ release in response to LppZ, ESAT-6, or CFP-10 was detected in parallel. The potential value of LppZ-specific IgA for TB and LTBI screening was also evaluated.

## Materials and methods

### Study populations

TB patients (*N* = 125), LTBI individuals (*N* = 92), and HCs (*N* = 165) were included in the study. TB patients were in-patients from Shanghai Public Health Clinical Center (Shanghai, China). TB was confirmed based on medical history, chest radiograph (X-ray and CT), acid-fast bacilli (AFB) smear or sputum culture. All the patients were both HIV-negative and HBV-negative. TB patients enrolled in the follow-up study were out-patients undergoing conventional anti-TB chemotherapy from Ruijin Hospital affiliated to Shanghai Jiao Tong University School of Medicine. Thirty-one onset patients were recruited at the starting point of the treatment, among them nine patients fulfilled the criteria of our study with the collection of samples at all-time points. Those who were older than 80 years old (*N* = 1), had virus infection (*N* = 1), or other diseases (*N* = 4) including diabetes, thrombocytopenia, etc., were excluded from this study. Sixteen individuals dropped out of the study before the end of the treatment. All TB patients had signed voluntary informed consent before being enrolled in this study.

LTBI individuals and HCs were from healthy blood donors undergoing annual physical examination in Ruijin Hospital (Shanghai, China). All healthy donors had no medical history or disease symptoms. LTBI individuals were defined as those with ≧6 spot forming units (SFUs) in T-SPOT assay. All individuals involved in this study were adults who had been vaccinated with the BCG Shanghai strain (Shanghai Institute of Biological Products Co., Ltd., Shanghai, China) during childhood. This study was approved by the Ethical Committee of Shanghai Jiao Tong University School of Medicine.

### Preparation of *M. tb* antigens

The recombinant plasmid pMRLB.54 containing gene *rv3006* (Protein LppZ) from *M. tb* (GeneBank accession No. NR-13304) was obtained from NIH Biodefense and Emerging Infection Research Resources Repository (NIAID, NIH, USA). The recombinant-expressing plasmids were transformed to *Escherichia coli* BL21 (DE3) and LppZ protein was induced for 3–4 h with 0.5 mM isopropyl β-D-1-thiogalactopyranoside (IPTG) (Beyotime, Jiangsu, China) at 30°C. Inducibly expressed protein was purified from bacteria through affinity chromatography with Ni-NTA His-Bind Resin (Qiagen, Duesseldorf, Germany). Lipopolysaccharide (LPS) was removed from purified LppZ protein by using Triton X-114 two-phase separation for at least six cycles (Jensen et al., 2008). In brief, Triton X-114 (Sigma, MO, USA) was added to the purified protein at the final concentration of 1% and incubated for 30 min at 4°C with constant agitation, followed by incubation for 10 min at 37°C and centrifugation at 16,000 × *g* for 10 min at 25°C. The remaining Triton X-114 was removed by ultrafiltration against PBS. The amount of LPS was measured by Tachyleus Amebocyte Lysate Kit (Gulangyu, Xiamen, China). Those preparations containing LPS lower than 0.10–0.15 EU/mL (which is the FDA endotoxin limit for drugs) were subjected to further experiments. Protein concentration was detected by BCA Protein Assay Kit (Thermo Fisher, MA, USA).

### SDS-PAGE and western blot

The purity of recombinant LppZ protein was determined by 12% sodium dodecyl sulfate polyacrylamide gel electrophoresis (SDS-PAGE) (Tanon, Shanghai, China) followed by Coomassie brilliant blue staining and further confirmed by Western blotting using anti-6 × His tag antibody (Abcam, Hong Kong, China) or human plasma (including TB patient, LTBI individual, HC and disease control). LppZ protein was electro-transferred to polyvinylidene fluoride (PVDF) membrane (Millipore, MA, USA). Human plasma was diluted at 1:200 ratio in the immunostaining assay. Horseradish peroxidase (HRP)-conjugated goat anti-mouse IgG (Cwbio, Beijing, China) and HRP-conjugated goat anti-human IgA (Cwbio) were used as secondary antibodies. The ECL solution (Millipore) was used to visualize LppZ. The membranes were scanned on Tanon 5200S Chemiluminescence Imaging System (Tanon).

### Peptide synthesis

Nineteen peptides were synthesized by Sangon Biotech (Shanghai, China). These peptides were 20 amino acid residues in length and covered the entire sequences of ESAT-6 or CFP-10 with overlapping 10-amino-acid residues.

### Peripheral blood mononuclear cell isolation and plasma collection

Whole blood (10 mL) was collected in tubes containing ethylene diamine tetraacetic acid (EDTA). Peripheral blood mononuclear cells (PBMCs) were isolated by Ficoll-hypaque density gradient centrifugation with Lymphoprep^TM^ solution (AXIS-SHIELD Poc AS, Oslo, Norway) according to the manufacturer's recommendation. The plasma was collected and stored at −80°C. The mononuclear cell layer was carefully transferred to a new 15 mL conical tube and washed twice with RPMI 1640 medium (GIBCO, NY, USA) by centrifuging at 486 × *g* for 10 min at room temperature. PBMCs were re-suspended at a concentration of 2.5 × 10^6^/mL in RPMI 1640 culture medium containing 10% fetal bovine serum (FBS) (Millipore), 100 units/mL penicillin (GIBCO), and 100 μg/mL streptomycin (GIBCO).

### Determination of LppZ-specific IgA in plasma

LppZ-specific IgA levels in human cohorts were determined by homemade enzyme-linked immunosorbent assay (ELISA). Briefly, 96-well polystyrene plates (Corning, NY, USA) were coated with 1 μg/mL purified LppZ protein diluted in bicarbonate/carbonate coating buffer (15 mM Na_2_CO_3_ and 35 mM NaHCO_3_, pH 9.6) and incubated at 4°C overnight. After washing three times with PBST (PBS containing 0.05% Tween-20; Sangon Biotech), the wells were blocked with 200 μL/well buffer G (PBS containing 1% pork gelatin; Sigma) at 37°C for 1 h. Human plasma (100 μL/well, 1:100 diluted in buffer G) was added to the wells and incubated at 37°C for 1 h. After washing three times with PBST, the wells were incubated with HRP-conjugated goat-anti-human IgA (SouthernBiotech, AL, USA) working solution (100 μL/well, diluted in buffer G) at 37°C for 1 h. Tetramethylbenzidine (TMB) (BD Bioscience, CA, USA) solution (100 μL/well) was added and the plates were incubated at room temperature in the dark for 15 min. The reaction was stopped by adding 1 M H_2_SO_4_ (50 μL/well). The absorbance at 450 nm was detected within 5 min by PowerWaveXS2 microplate spectrophotometer (BioTek Instruments, Inc., VT, USA).

### Interferon-gamma (IFN-γ) release assay

Antigen-specific IFN-γ releasing level was determined by using enzyme-linked immunospot (ELISPOT) assays according to the manufacturer's instructions (U-CyTech, Utrecht, Netherlands). Briefly, 96-well PVDF plates (Millipore) were coated with anti-human IFN-γ coating antibody overnight at 4°C. The wells were blocked for 1 h at 37°C then 2.5 × 10^5^ PBMCs in 100 μL culture medium were plated in each well and stimulated with ESAT-6 or CFP-10 peptide pool (2 μg/mL per peptide), or 20 μg/mL tuberculin PPD (Statens Serum Institute, Copenhagen, Denmark) or purified LppZ protein (10 μg/mL). Culture medium served as a negative control and 2.5 μg/mL phytohemagglutinin (PHA) (Sigma) was used as a positive control. After 20-h incubation at 37°C, the plates were incubated with biotin-labeled detection antibody at 37°C for 1 h and subsequently HRP-conjugated streptavidin working solution for another 1 h. AEC substrate solution was added to each well for 30 min in the dark at room temperature. Color development was stopped by thoroughly rinsing both sides of the PVDF membrane with demineralized water. The plates were dried in the dark at room temperature. The spots were counted by ELISPOT BioReader-4000 (BIO-SYS GmbH, Karben, Germany). The number of antigen-specific IFN-γ-producing cells was calculated based on spot-forming units (SFUs) per 2.5 × 10^5^ PBMCs after deducting the background SFUs detected by the paired negative control wells. Individuals that did not meet the criteria of positive (SFU > 5) or negative (SFU < 20) controls were ruled out.

### Statistical analysis

All the data are shown as mean ± S.E.M. Statistical analyses were performed by using GraphPad Prism 5.0 software (GraphPad Software Inc., CA, USA). Statistical differences were assessed by the unpaired *t*-test for the data with Gaussian distribution and by Mann-Whitney test for those with non-Gaussian distribution. Receiver operator characteristic (ROC)-curves were constructed by plotting the true positive rate (sensitivity) against the false-positive rate (1-specificity). Areas under curve (AUC)-values were calculated along with 95% confidence intervals (95% CI). In order to reduce false positive and false negative rates, optimal cutoff values of antigen-specific IgA and antigen-specific IFN-γ releasing levels were determined when the Youden index (YI)-value was maximal according to ROC-curves.

## Results

### Study subjects

TB patients (*N* = 125), LTBI individuals (*N* = 92), and HCs (*N* = 165) were involved in this study (Table 1). Among TB patients (age: 51.88 ± 19.95; Female/Male: 19/106) there were pulmonary tuberculosis (PTB) (*N* = 63), extra pulmonary tuberculosis (EPTB) (*N* = 19), and other tuberculosis patients (defined as PTB patients who additionally had EPTB; *N* = 43). Among the 63 PTB patients, 31 (49.21%) were sputum smear positive, and 30 (47.62%) were sputum culture positive. Nineteen of the PTB patients (30.16%) were diagnosed with pulmonary cavitation.

**Table 1:** Age and Gender of TB, LTBI, and HC subjects.

	**TB**	**LTBI**	**HC**
N	125	92	165
Gender (F/M)	19/106	45/47	82/83
Age (mean ± *SD*)	51.88 ± 19.95	49.82 ± 15.40	39.48 ± 11.93

LTBI subjects were discriminated from healthy volunteers by using IFN-γ ELISPOT assay in 257 healthy volunteers. There were 92 subjects with ≧6 SFUs after stimulation with synthesized ESAT-6 peptide pool (E6p) or CFP-10 peptide pool (C10p) and they were defined as LTBI individuals (age: 49.82 ± 15.40; Female/Male: 45/47). The percentage of LTBI in healthy donors (35.8%) was close to the estimation of around 33% reported by WHO (2015). HCs (*N* = 165; age: 39.48 ± 11.93; Female/Male: 82/83) were defined as those with SFUs < 6.

In addition, a follow-up study was performed in TB patients undergoing first-line anti-TB treatment (Table 2) that included isoniazid (INH), rifampicin (RFP), ethambutol (EMB), and pyrazinamide (PZA), for at least 6 months according to the national tuberculosis control program (NTP) in China (Huang et al., 2016). Among nine follow-up TB patients (age: 32.56 ± 12.47; Female/Male: 2/7) investigated in this study, four were sputum culture positive (44.4%) and two were sputum smear positive (22.2%). The proportion of patients without *M. tb* cavitation substantially increased from 66.67% before the treatment to 77.78% at the second month and 100% at the sixth month after anti-TB treatment.

**Table 2:** Follow-up of TB patients undergoing anti-TB treatment.

	***N***	**Gender (F/M)**	**Age (mean ± *SD*)**	**Sputum smear *N* (%)**	**Sputum culture *N* (%)**	**Cavity conversion**, ***N*** **(%)**		
						**Month 0**	**Month 2**	**Month 6**
Follow-up TB	9	2/7	32.56 ± 12.47	2 (22.2)	4 (44.4)	6(66.67)	7(77.78)	9(100)

### Preparation and identification of lipoprotein Z

**Figure 1:**
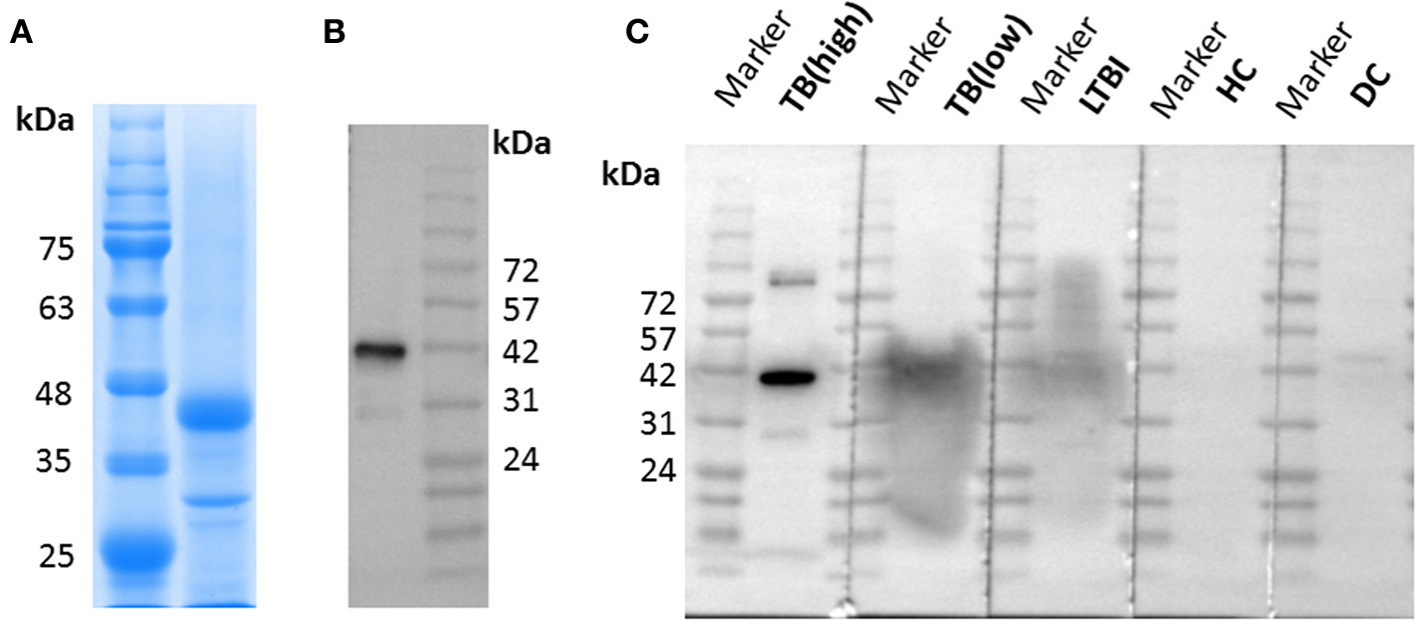
Expression and identification of LppZ protein. **(A)** SDS-PAGE analysis of purified LppZ protein expressed in *E. coli*. LppZ was purified by Ni-NTA column and applied to 12% SDS-PAGE. LppZ protein was visualized through staining with Coomassie brilliant blue. **(B)** Western blotting of purified LppZ protein. Purified His-tagged LppZ was detected with HRP conjugated anti-6 × His tag mouse antibody. **(C)** Western blotting of purified LppZ protein. Purified LppZ was detected with plasma from two TB patients, one LTBI individual, one HC, and one disease control (DC).

LppZ protein was expressed in *E. coli* BL21 (DE3) and purified by Ni-NTA His-Bind Resin. The purity was identified by using SDS-PAGE which showed the expected molecular weight of ~38 kDa (Figure 1A, Supplementary Figure 1), and further confirmed with anti-6 × His tag antibody (Figure 1B, Supplementary Figure 1). In addition, purified LppZ protein was blotted with human plasma from two TB patients (one with high LppZ-specific IgA-value and one with low LppZ-specific IgA-value according to the results from ELISA), one LTBI individual, one HC, and one non-LTBI lung cancer patient as a disease control (DC). LppZ-specific IgA could indeed be detected in the plasma of the *M. tb*-infected individuals, including TB patients and LTBI individual, whereas it was hardly detectable in the plasma of *M. tb* non-infected subjects including HC and DC individuals (Figure 1C). The results from Western blotting with human plasma indicated that LppZ-specific IgA was readily detectable in TB and LTBI individuals but minimal in *M. tb* non-infected individuals.

### TB patients exhibit dramatic LppZ-specific IgA responsiveness in the periphery

**Figure 2:**
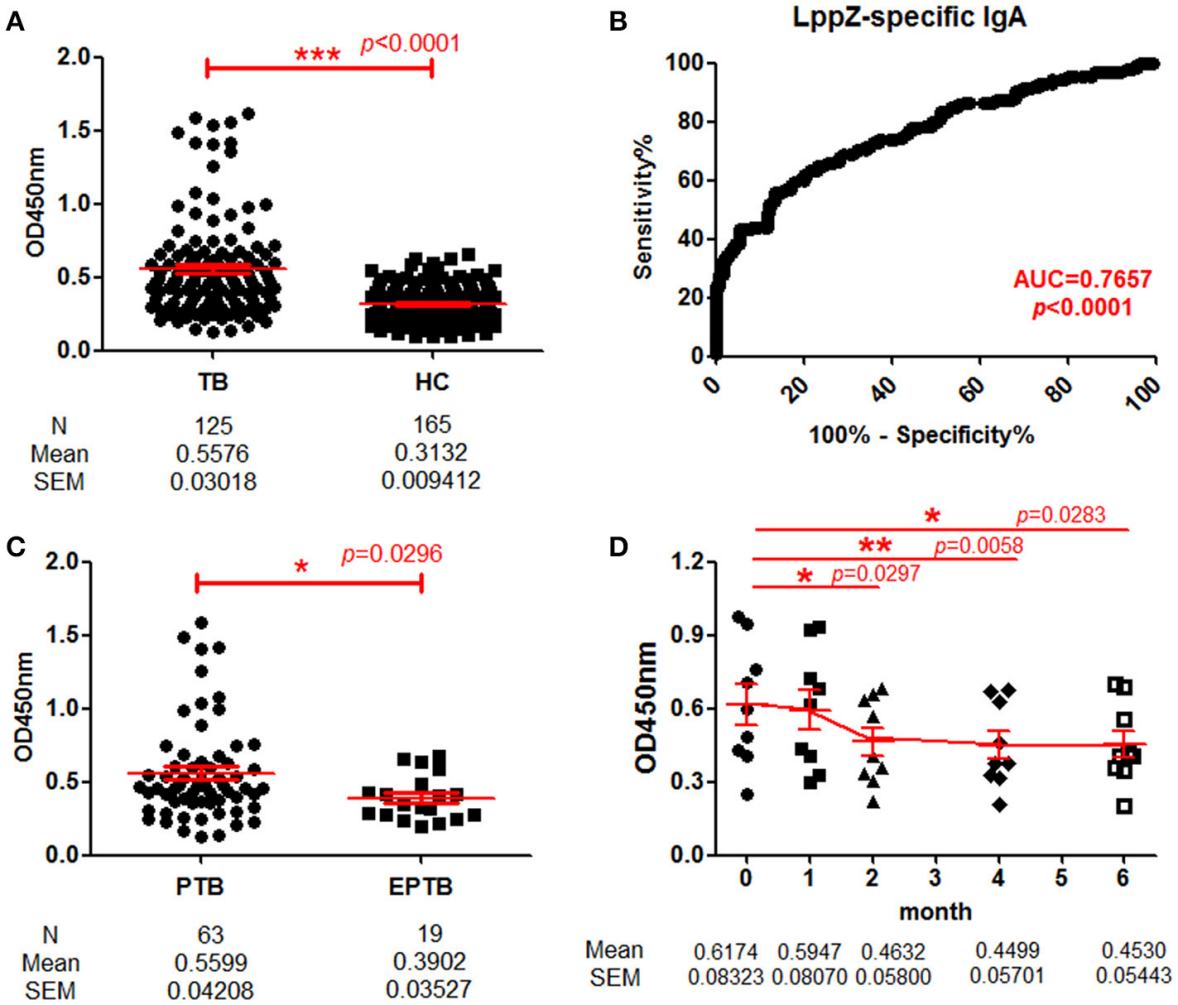
Determination of LppZ-specific IgA level in TB patients. **(A)** Comparison of optical density (OD_450_)-values of LppZ-specific IgA in the plasma between TB patients (*N* = 125) and HCs (*N* = 165). The *P-value* was calculated using Mann-Whitney test. **(B)** Receiver operating characteristic (ROC)-curve for LppZ-specific IgA to discriminate TB (*N* = 125) from HCs (*N* = 165). Area under curve (AUC) was 0.7657 (95% CI, 0.7096–0.8218). **(C)** Comparison of OD_450_-values of LppZ-specific IgA in pulmonary TB (PTB) patients (*N* = 63) and extra-pulmonary TB (EPTB) patients (*N* = 19). The *P-value* was calculated using Mann-Whitney test. **(D)** Cumulative OD_450_-values of LppZ-specific IgA in TB patients (*N* = 9) over the course of anti-TB treatment, including the time of diagnosis (month 0) and the time points at month 1, 2, 4, 6 during the treatment. The *P-value* was calculated using paired *t*-test. ^*^0.01 < *p* < 0.05, ^**^0.001 < *p* < 0.01, ^***^p < 0.001.

To quantitatively determine LppZ-specific IgA levels in the plasma, a homemade ELISA was performed and LppZ-specific IgA levels were compared between TB patients (*N* = 125) and HCs (*N* = 165). The LppZ-specific IgA level was significantly higher in plasma from TB than from HCs (*p* < 0.0001) (Figure 2A) and dramatically higher from PTB patients (*N* = 63) than from EPTB patients (*N* = 19) (*p* = 0.0296) (Figure 2C). In addition, the LppZ-specific IgA level was monitored in the follow-up study over the course of anti-TB treatment, including at the time of diagnosis (month 0) and at months 1, 2, 4, and 6. Compared to month 0, LppZ-specific IgA levels had decreased significantly at months 2, 4, and 6 after the treatment (*p* = 0.0297, *p* = 0.0058, *p* = 0.0283, respectively) (Figure 2D). However, even at month 6 LppZ-specific IgA level was still higher than that in HCs. We also determined the AUC-value of LppZ-specific IgA based on a ROC-curve and this reached 0.7657 (*p* < 0.0001) (Figure 2B).

Thus, the LppZ-specific IgA level was significantly higher in TB patients and more dramatically in PTB patients. With the decline of LppZ-specific IgA levels during anti-TB treatment, there was a presumptive correlation with the bacillary load.

### Negative correlation between LppZ-specific IgA level and cellular immune response

**Figure 3:**
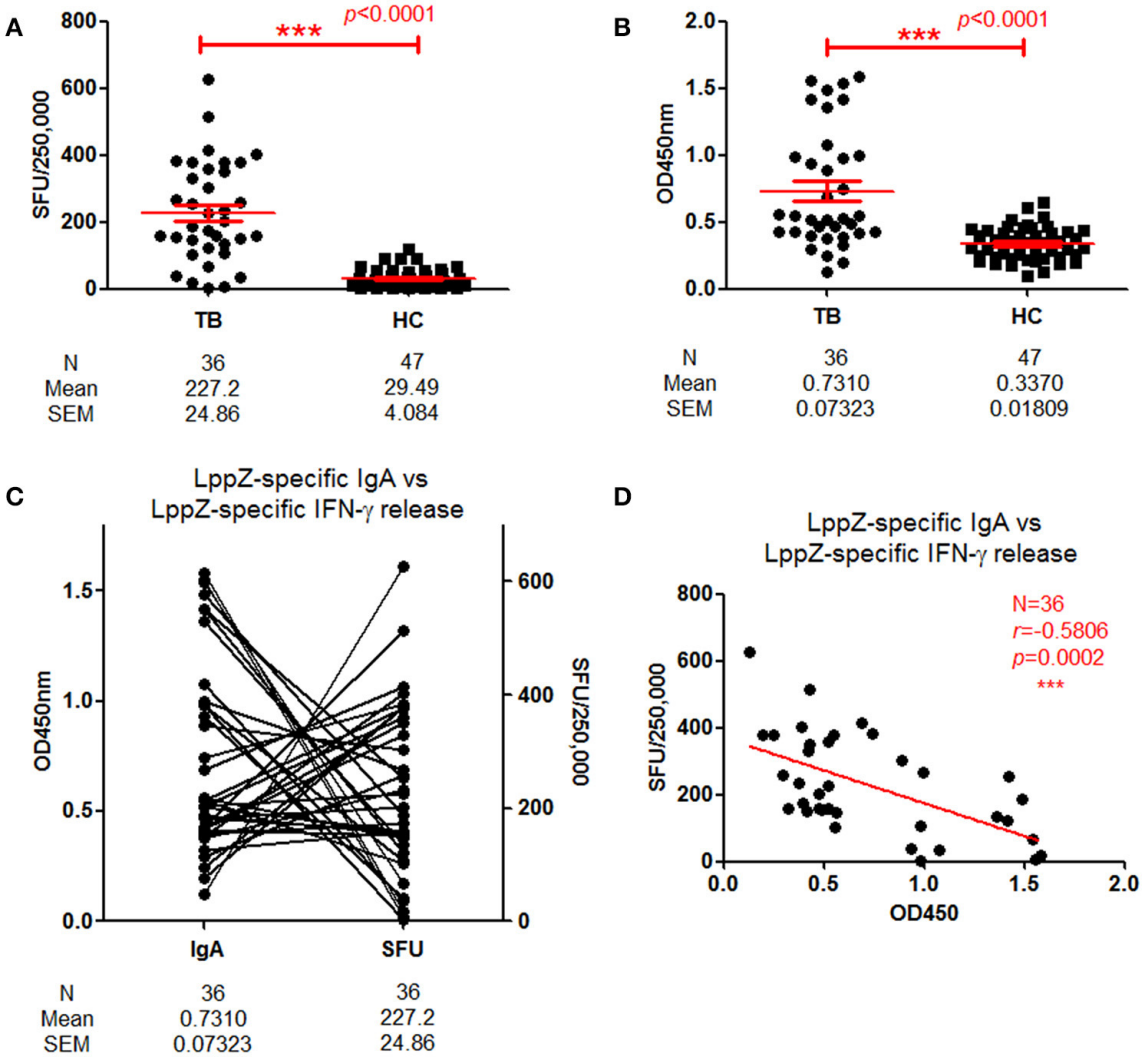
Correlation between LppZ-specific IgA and LppZ-specific IFN-γ releasing level in TB patients. **(A)** LppZ-specific IFN-γ releasing cell numbers in PBMCs from TB patients (*N* = 36) and HCs (*N* = 47). The *P-value* was calculated by using Mann Whitney test. **(B)** OD_450_-values of LppZ-specific IgA in TB patients (*N* = 36) and HCs (*N* = 47). The *P-value* was calculated by using Mann Whitney test. **(C)** Complementarity analysis between LppZ-specific IgA and LppZ-specific IFN-γ releasing level in TB patients (*N* = 36). Each line represented one individual. **(D)** Correlation analysis between LppZ-specific IgA and LppZ-specific IFN-γ releasing level in TB patients (*N* = 36). The correlation coefficient *r* (*r* = −0.5806) and the *P-value* (*p* = 0.0002) were calculated using the Spearman rank test. SFU: Spot forming unit. ^***^p < 0.001.

These results indicated that LppZ was an immune dominant antigen capable of inducing strong IgA humoral response. Our previous work indicated that it could also trigger specific cellular immune responses. The relationship between LppZ-specific humoral and cellular responses was further analyzed. LppZ-specific release of IFN-γ from PBMCs of TB (*N* = 36) and HC subjects (*N* = 47) was assayed in parallel. Similar to our previous results, the frequency of cells releasing IFN-γ in response to LppZ was significantly higher in the TB group than in HCs (*p* < 0.0001) (Figure 3A) and this was accompanied by higher LppZ-specific IgA level in the TB group than that in HCs in the same cohorts (*p* < 0.0001) (Figure 3B). Analysis of the correlation between LppZ-specific IFN-γ SFUs and LppZ-specific IgA-values in the TB group indicated that those patients with higher LppZ-specific IgA levels exhibited lower LppZ-specific IFN-γ SFUs, or *vice versa* (Figure 3C). According to Spearman rank test, there was a significant negative correlation between LppZ-specific IgA levels and LppZ-specific IFN-γ releasing levels in TB patients (*r* = −0.5806, *p* = 0.0002) (Figure 3D).

These data demonstrated that LppZ-specific IgA and IFN-γ releasing levels were both significantly higher in TB patients than in HCs. However, either the IgA humoral or the IFN-γ cellular immune response to LppZ was dominant in individual TB patients.

### Determination of LppZ-specific IgA levels in LTBI individuals

**Figure 4:**
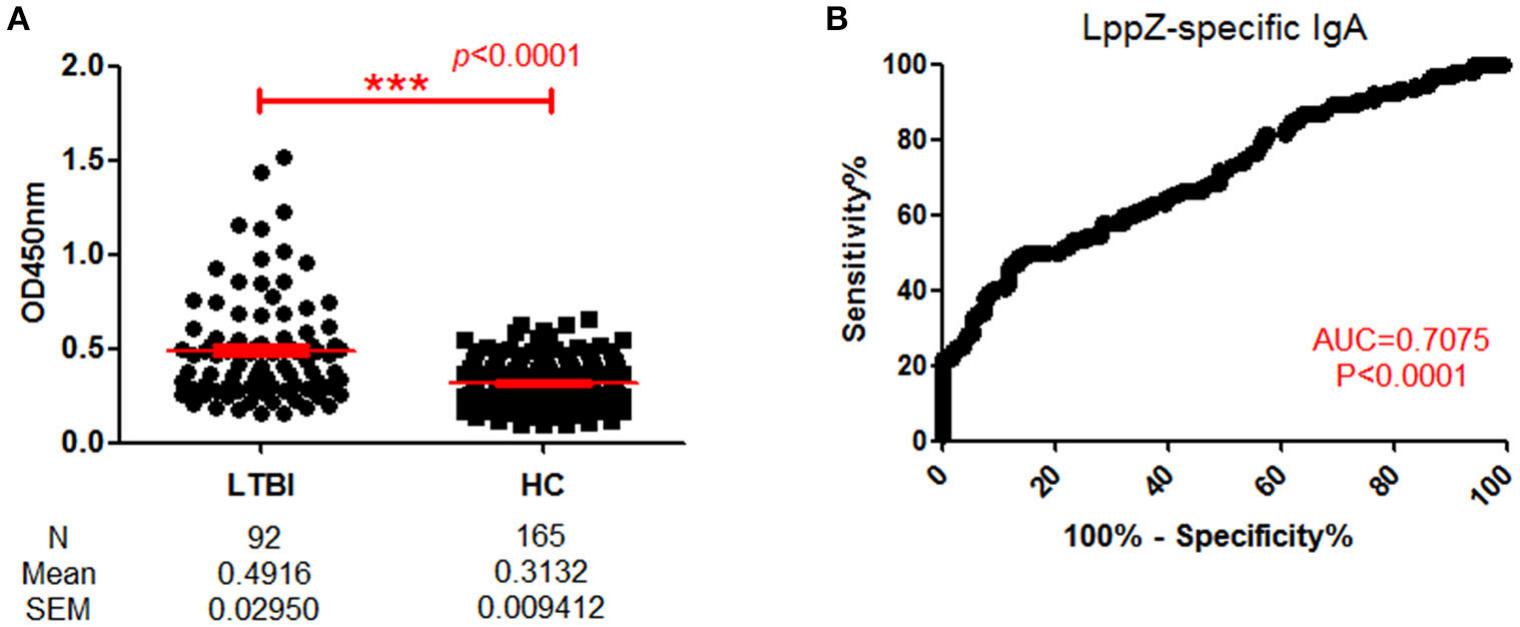
Determination of LppZ-specific IgA level in LTBI individuals. **(A)** OD_450_-values of LppZ-specific IgA in LTBI (*N* = 92) and HCs (*N* = 165). The *P-value* was calculated using Mann Whitney test. **(B)** ROC-curve for LppZ-specific IgA to discriminate LTBI individuals from HCs. The *P-value* was calculated using Mann Whitney test. AUC was 0.7075 (95% CI, 0.6396–0.7754). ^***^p < 0.001

Since LppZ induced strong IgA responses when there was active TB pathogenesis and a detectable *M. tb* bacilli burden, it was questionable whether the response would also be found under latent TB infection (LTBI). When LppZ-specific IgA level was compared between LTBI individuals (*N* = 92) and HCs (*N* = 165), the LppZ-specific IgA level in LTBI individuals was significantly higher than that in HCs (*p* < 0.0001) (Figure 4A). The ROC-curve showed an AUC-value of 0.7075 (*p* < 0.0001) (Figure 4B). These results further demonstrated that LppZ is a dominant mycobacterial antigen, inducing strong IgA responses even in latent infection by the *M. tb* pathogen.

### Inclusion of the LppZ-specific IgA assay increases the sensitivities of ESAT-6 and CFP-10-specific IFN-γ release assays for TB and LTBI screening

Since the LppZ-specific IgA level was elevated both in TB patients and LTBI individuals, the potential utility of this finding was investigated. Based on the cutoff value of LppZ-specific IgA in the TB group (0.375), which was determined based on ROC-curve analysis and maximal YI, assay of LppZ-specific IgA alone had a sensitivity of 86.11% and specificity of 72.12% in TB patients (*N* = 36) (Table 3). However, when the IgA results were coordinated with ESAT-6 or CFP-10 specific IFN-γ releasing levels (Supplementary Figure 2), the diagnostic sensitivities of the ESAT-6 and CFP-10-specific IFN-γ release assays for TB patients were substantially improved from 86.11 to 100% and 88.89 to 100%, respectively, and the specificities remained at 72.12 and 72.12% (Table 3). In the LTBI cohort (Supplementary Figure 3), the cutoff value of LppZ-specific IgA in LTBI was 0.4385. When this assay was combined with ESAT-6 or CFP-10-specific IFN-γ releasing assays in screening LTBI, the sensitivities were elevated from 80.36 to 83.93% and from 57.14 to 69.64%, with the specificities of 85.45 and 84.85%, respectively (Table 4).

**Table 3:** The sensitivity and specificity of LppZ-specific IgA assay and ESAT-6 or CFP-10-specific IFN-γ release assay for TB diagnosis^#^.

	**Cutoff value**	**Sensitivity (%) (95% CI)**	**Specificity (%) (95% CI)**
LppZ-specific IgA	0.375	86.11 (70.50–95.33)^***^	72.12 (64.62–78.81)
ESAT-6 specific IFN-γ releasing	5	86.11 (70.50–95.33)^***^	100 (97.79–100.0)
CFP-10 specific IFN-γ releasing	7	88.89 (73.94–96.89)^***^	99.39 (96.67–99.98)
LppZ-specific IgA + ESAT-6 specific IFN-γ releasing	–	100 (–)	72.12 (–)
LppZ-specific IgA + CFP-10-specific IFN-γ releasing	–	100 (-)	72.12 (–)

*** *p < 0.001*.

# *TB: N = 36; HC: N = 165*.

*CI, confidence intervals*.

**Table 4:** The sensitivity and specificity of LppZ-specific IgA assay and ESAT-6 or CFP-10-specific IFN-γ release assay for LTBI screening^#^.

	**Cutoff value**	**Sensitivity (%) (95% CI)**	**Specificity (%) (95% CI)**
LppZ-specific IgA	0.4385	32.14 (20.29–45.96)^***^	85.45 (79.13–90.45)
ESAT-6 specific IFN-γ releasing	5	80.36 (67.57–89.77)^***^	100 (97.79–100.0)
CFP-10 specific IFN-γ releasing	5	57.14 (43.22–70.29)^***^	99.39 (96.67–99.98)
LppZ-specific IgA + ESAT-6 specific IFN-γ releasing	–	83.93 (–)	85.45 (–)
LppZ-specific IgA + CFP-10-specific IFN-γ releasing	–	69.64 (–)	84.85 (–)

*** *p < 0.001*.

# *LTBI: N = 56; HC: N = 165*.

*CI, confidence intervals*.

Therefore, although the assay of LppZ-specific IgA is not good enough on its own for clinical application, it can increase the sensitivities of ESAT-6 or CFP-10-specific IFN-γ release assays in TB and LTBI screening, suggesting the potential value of LppZ-specific IgA as an adjunctive screening biomarker.

### Individuals with enhanced LppZ-specific IgA levels in the PPD^+^ healthy group

**Figure 5:**
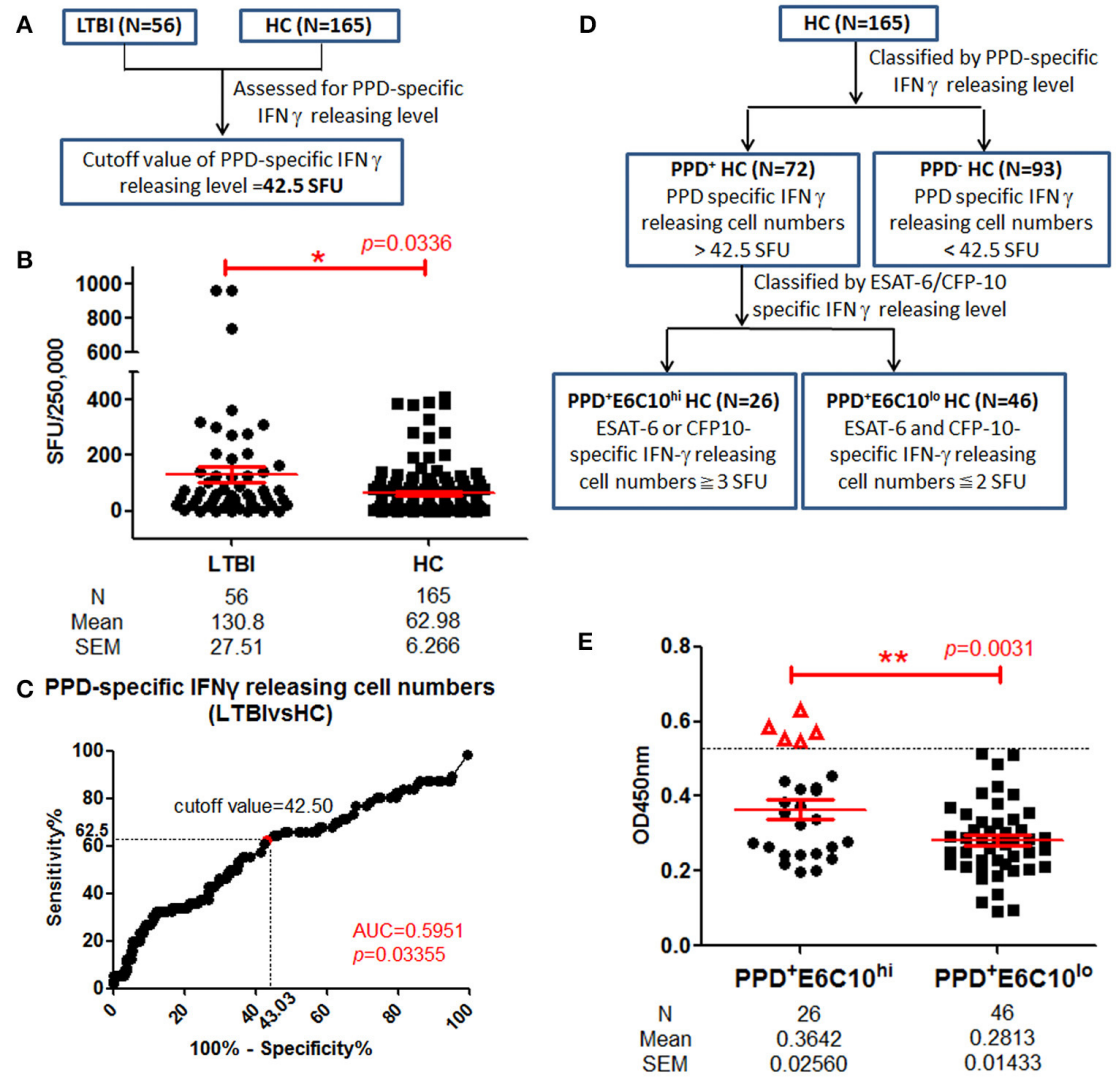
LppZ-specific IgA level in PPD^+^ HC groups. **(A)** Flow diagram to define the cutoff value of PPD-specific IFN-γ releasing levels in HCs. **(B)** PPD-specific IFN-γ releasing cell numbers in LTBI individuals (*N* = 56) and HCs (*N* = 165). The *P-value* was calculated using Mann Whitney test. **(C)** ROC-curve for PPD-specific IFN-γ releasing cell numbers between LTBI individuals (*N* = 56) and HCs (*N* = 165). The cutoff value was calculated using maximal Youden Index. **(D)** Flow diagram to define PPD^+^E6C10^hi^ and PPD^+^E6C10^lo^ HCs. **(E)** LppZ-specific IgA levels of PPD^+^E6C10^hi^ HCs (*N* = 26) and PPD^+^E6C10^lo^ HCs (*N* = 46). The *P*-value was calculated using Mann Whitney test. Δ represents those with higher LppZ-specific IgA level than cutoff value (0.4585). PPD^+^ HCs: HC individuals with PPD-specific IFN-γ releasing cell numbers > cutoff value (42.5 SFU); PPD^+^E6C10^hi^ HCs: PPD^+^HCs with ESAT-6 or CFP-10-specific IFN-γ releasing cell numbers ≧ 3 SFU; PPD^+^E6C10^lo^ HCs: PPD^+^HCs with ESAT-6 and CFP-10-specific IFN-γ releasing cell numbers ≦ 2 SFU. ^*^0.01 < *p* < 0.05, ^**^p < 0.01.

Currently, LTBI screening in healthy populations relies mainly on detecting a positive IGRA result in individuals with no clinical symptoms. However, this still cannot exclude the possibility that there exist some undetected leaky LTBI individuals among HCs. In our study, we have performed the IGRA assay in 221 healthy volunteers among whom 56 individuals were identified as LTBI based on SFUs-values≧6 in tests with ESAT-6 or CFP-10. Because PPD has an auxiliary role in TB diagnosis, PPD-specific IFN-γ releasing levels were also assayed in LTBI individuals (*N* = 56) and HCs (*N* = 165) in parallel (Figure 5A). Increased PPD-specific IFN-γ releasing levels were observed in LTBI individuals (Figure 5B). On ROC-curve analysis the cutoff value of the PPD-specific IFN-γ releasing level was calculated as 42.5 SFUs using maximal YI-value (Figure 5C) and this divided HCs into PPD^+^ (*N* = 72) and PPD^−^ (*N* = 93) groups. As shown in Figure 5D, the PPD^+^ HC group was further classified into two groups: a PPD^+^E6C10^hi^ HC group (*N* = 26), defined as PPD^+^ HCs with ESAT-6 or CFP-10-specific IFN-γ releasing cell numbers ≧3 and a PPD^+^E6C10^lo^ HC group (*N* = 46), defined as having ESAT-6 and CFP-10-specific IFN-γ releasing cell number ≦2. The LppZ-specific IgA level in the PPD^+^E6C10^hi^ HCs was significantly higher than that in the PPD^+^E6C10^lo^ HCs (*p* = 0.0031) (Figure 5E). What is more, there existed 5 PPD^+^E6C10^hi^ individuals (highlighted as “Δ” in Figure 5E) who had LppZ-specific IgA levels that were not only higher than the cutoff value (0.4585) but also higher than all PPD^+^E6C10^lo^ HC individuals. Considering that the assay of LppZ-specific IgA level remarkably increased the sensitivity of ESAT-6 or CFP-10-specific IGRA assay in LTBI screening, it can be speculated that these 5 PPD^+^E6C10^hi^ HCs might be high-risk LTBI candidates.

## Discussion

Nowadays, lipoproteins in *M. tb* are being increasingly studied in respect to roles in both virulence and immunity (Rezwan et al., 2007; Buddelmeijer, 2015). They display strong immunogenicity that is probably related to their prevalent distribution and abundant expression in mycobacterial pathogens. So far, some of the lipoproteins have been identified as potential biomarker proteins for TB discrimination. For instance, PstS1 (Rv0934), also known as 38kDa protein, shows great immunogenicity (Målen et al., 2008) with the ability to induce B and T cell responses upon *M. tb* infection (Harboe and Wiker, 1992). Moreover, 38kDa protein has been recommended to be used in TB sero-diagnosis (Senol et al., 2009). LppZ, as investigated here, also exhibits strong immunogenicity in both humoral and cellular aspects and in both TB and LTBI population. More interestingly, elevated anti-LppZ IgA responses are more evident than IgG responses in TB and LTBI (data not shown). This is probably due to the fact that *M. tb* infection occurs mainly through the respiratory tract. Results from the comparison between PTB and EPTB cases, where *M. tb* is considered to have less involvement of the respiratory tract in EPTB, also support the conclusion that TB pathogenesis triggers strong mucosal immunity leading to the induction of secretory IgA. In addition, the substantial decrease in LppZ-specific IgA levels in the follow-up TB patients accompanies the amelioration of the disease at month 2 after anti-TB treatment. Since most of the patients become bacilli negative at month 2 when they finish the intensive phase of anti-TB treatment, our results strongly suggest that the generation of IgA-mediated mucosal immunity is largely due to *M. tb* infection. Similar results are observed in IgA reactivity to glycolipid antigen after 2 and 6 months during anti-TB therapy, suggesting a potential role for antigen-specific IgA in monitoring pulmonary TB treatment (Bezerra et al., 2009). However, we did not see strong correlation between LppZ-specific IgA levels and disease severity in PTB patients, as indicated by smear positivity, culture positivity and cavitation (Supplementary Figure 4).

Our findings demonstrate that LppZ is of high immunogenicity upon *M. tb* infection and several studies indicate that both humoral and cellular immune responses are involved in protection against *M. tb* infection (Falla et al., 1991; Mattos et al., 2010; Scriba et al., 2017). The observation that high levels of *M. tb*-specific antibodies could be detected without TST reactivity suggests that *M. tb*-specific antibodies could be elevated in the absence of cellular immune response (Bothamley et al., 1992; Sousa et al., 1997). In our study, LppZ exhibits both higher cellular and higher IgA responses in TB patients when compared to HCs. Moreover, LppZ-specific IgA levels are negatively correlated to LppZ-specific IFN-γ releasing levels in TB patients. We therefore speculate that there might exist a dominant immunity pattern targeting LppZ antigen that differs between individuals upon *M. tb* infection; either humoral or cellular may be selected for dominance.

Because of the elevated LppZ-specific IgA in TB and LTBI cohorts, we intend to evaluate its potential in clinical application. The AUC-values of LppZ-specific IgA (0.7657 for TB diagnosis and 0.7075 for LTBI screening) theoretically imply its potential in discriminating *M. tb*-infected individuals from non-infected individuals. However, the LppZ-specific IgA assay has sensitivity and specificity that are too low to fulfill the criteria for either TB diagnosis or LTBI screening when compared with other available diagnostic methods (Tables 3, 4). Thus, the IgA-based test on its own seems unlikely to provide an adequate biomarker for TB and LTBI identification. Consequently we can consider what clinical application there might be for using such strong LppZ-specific IgA responses in TB in the future. At present, *M. tb*-specific ESAT-6 or CFP-10-based IGRA assays are widely used in distinguishing *M. tb*-infected from *M. tb* non-infected individuals (Pai et al., 2014). However, the specificity and sensitivity are still under question due to the different stages and situations of *M. tb* infection which arise. Recently, Li et al. (2017) demonstrated that higher sensitivity for TB diagnosis could be achieved when combining *M. tb* antigen Rv3615c with ESAT-6 and CFP-10 as stimulus. Another strategy for improvement is to add extra biomarkers in the assay. Here we show that the combination of measurement of LppZ-specific IgA together with assays of ESAT-6 or CFP-10 specific IFN-γ releasing levels could markedly raise the sensitivity in screening *M. tb*-infected individuals (Tables 3, 4). Thus, measurement of LppZ-specific IgA holds significant potential as an adjunctive biomarker for monitoring *M. tb* infection.

It has been well-acknowledged that both TST and IGRA assays can be used to determine the presence of *M. tb*-specific T cell responses (Young et al., 2009; Li et al., 2017). While the PPD-based TST does reveal the antigen-specific immune responses to previous infection (Morrison et al., 2008; Young et al., 2009), the IGRA assay reflects *M. tb* infection more specifically (Hill et al., 2007). For decades, LTBI individuals have been identified mainly on the basis of positive results in either TST or IGRA assays, despite the fact that both methods are deficient to some degree in sensitivity. For instance, a proportion of repeatedly TB-exposed healthy people never convert into positivity in TST assay (Joshi et al., 2006). ESAT-6/CFP-10 specific cellular responses mainly stand out for their extremely high specificity (usually over 90%) in diagnosing *M. tb* infection (van Pinxteren et al., 2000; Harada et al., 2004). By following the protocol of the assay kit manufacturer, subjects with ≧6 SFUs against synthesized ESAT-6 or CFP-10 peptide pools are screened out as LTBI individuals. This standard for screening LTBI individuals has been suspected to be not as sensitive as expected (Hill et al., 2007), indicating the existence of false-negative LTBI individuals during screening. In our study, we sub-grouped HCs based on PPD and ESAT-6/CFP-10 specific cellular responses. We speculated that subjects with high IFN-γ releasing levels in response to both PPD and ESAT-6/CFP-10 were more likely to have *M. tb* infection. Consistent with this, when LppZ-specific IgA level was compared between PPD^+^E6C10^hi^ (more than three SFUs upon E6C10 stimulation) and PPD^+^E6C10^lo^ (< 3 SFUs) HC subjects, it was shown that not only were the average levels of LppZ-specific IgA higher, but also there existed 5 HCs with higher LppZ-specific IgA levels in the PPD^+^E6C10^hi^ group (Figure 5E). The values of LppZ-specific IgA of five HCs were higher than those in PPD^+^E6C10^lo^ group. Considering the role of LppZ-specific IgA in indicating *M. tb* infection, we presume that those five individuals might be “leaky LTBI” individuals. Whether the assay of LppZ-specific IgA could be applied to increase the sensitivity of LTBI screening needs to be further investigated using cohort studies.

## Conclusions

In the present study, we report higher LppZ-specific IgA levels in both TB and LTBI groups when compared to HCs, indicating that LppZ is of high immunogenicity upon *M. tb* infection. What's more, combining assay of LppZ-specific IgA with that of ESAT-6 or CFP-10 specific IFN-γ releasing levels dramatically increases the sensitivities of TB and LTBI screening when compared to using ESAT-6 or CFP-10 specific IFN-γ releasing assay alone. Although sero-diagnosis is not recommended by WHO among standard diagnostic methods for TB (WHO, 2010), the high levels of LppZ-specific IgA in both TB and LTBI reveal a potential role in increasing the sensitivity of the present diagnostic methods. It may serve as a complementary biomarker for monitoring disease treatment and *M. tb* infection. Its potential in identifying leaky LTBI in PPD^+^ healthy populations needs to be further investigated through follow-up studies.

## Author contributions

YW and SL designed the experiments. JX, YC, YX conducted the experiments. YW, SL, GZ, JX, and YC analyzed the data. YX, QC, and SL collected the samples and clinical data. PJ, SW, Y-jX, and YL contributed reagents, materials, analysis tools. YW, SL, and JX wrote the manuscript.

### Conflict of interest statement

The authors declare that the research was conducted in the absence of any commercial or financial relationships that could be construed as a potential conflict of interest.
