# Supplementary material for: Determination of Lipoprotein Z-Specific IgA in Tuberculosis and Latent Tuberculosis Infection
Source: Front Cell Infect Microbiol. 2017 Nov 30;7:495. doi: 10.3389/fcimb.2017.00495 (PMC5715530; doi:10.3389/fcimb.2017.00495)
Supplement: Supplementary file 1 [file DataSheet1.docx]

Supplementary Material

Determination of Lipoprotein Z-specific IgA in Tuberculosis and Latent Tuberculosis Infection

**Jia-ni Xiao^1#^, Yanqing Xiong^2#^, Yingying Chen^1#^, Yang-jiong Xiao^1^, Ping Ji^1^, Yong Li^1^, Shu-jun Wang^1^, Guo-ping Zhao^4^, Qi-jian Cheng^3^, Shui-hua Lu^2*^, Ying Wang^1*^**

*** Correspondence:**

Prof. Ying Wang
[ywang@sibs.ac.cn](mailto:ywang@sibs.ac.cn)

Prof. Shui-hua Lu
[lushuihua66@126.com](mailto:lushuihua66@126.com)


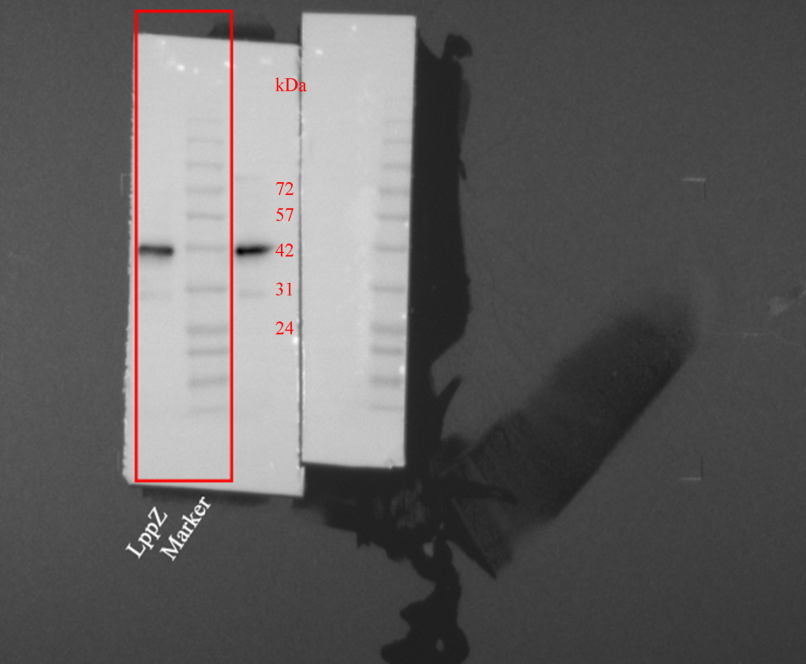


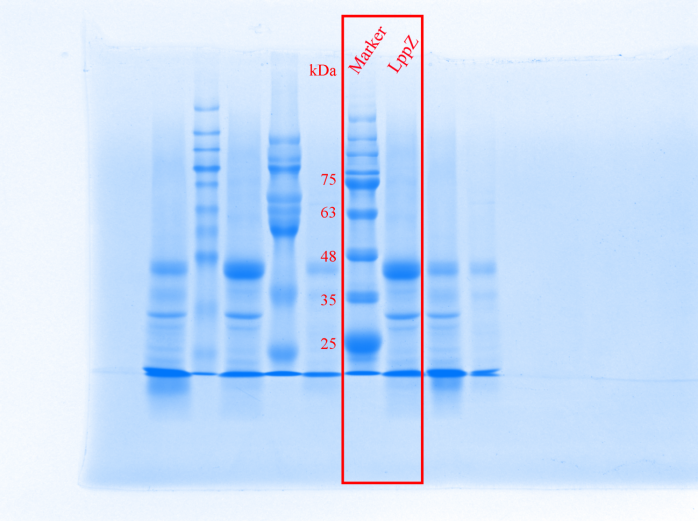


**Supplementary Figure 1 Original image of Identification of LppZ protein by SDS-PAGE (left) and Western blotting (right)**


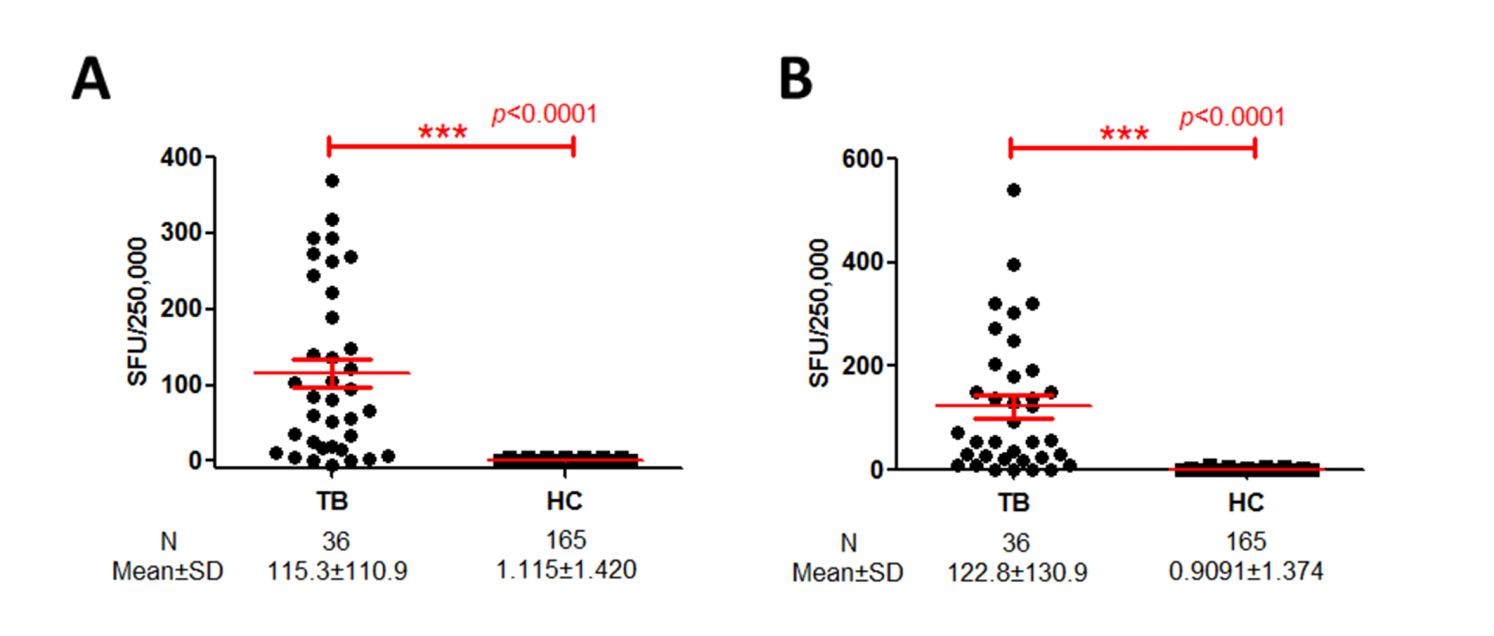


**Supplementary Figure 2 Comparison of ESAT-6 or CFP-10-specific IFN-γ releasing levels between TB and HC groups**

Scatter plots including TB patients (N=36) and HCs (N=165) showing antigen-specific IFN-γ releasing cell numbers to ESAT-6 (*p* < 0.0001) (**A**) and CFP-10 (*p* < 0.0001) (**B**). The *P-value* was calculated using Mann Whitney test. ***: *p* < 0.001


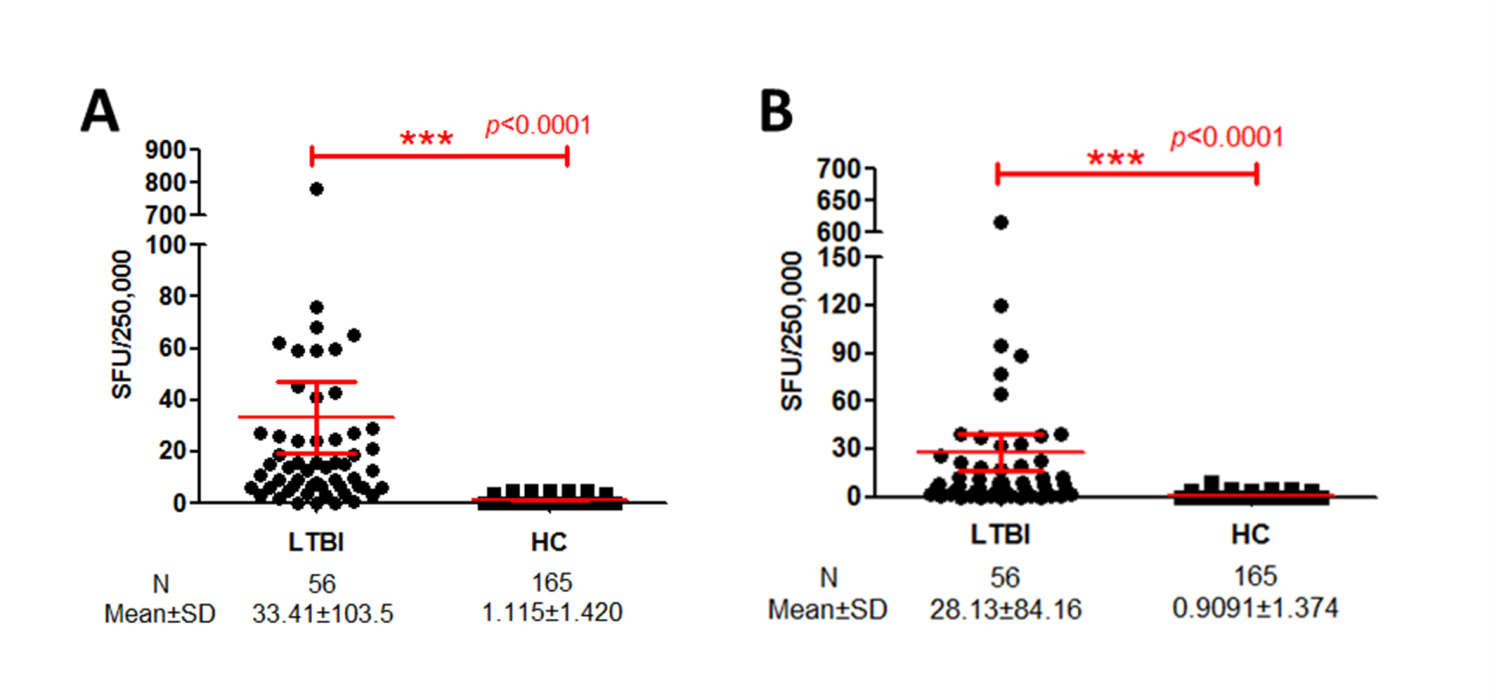


**Supplementary Figure 3 Comparison of ESAT-6 or CFP-10-specific IFN-γ releasing levels between LTBI and HC groups**

Scatter plots including LTBI individuals (N=56) and HCs (N=165) showing antigen-specific IFN-γ releasing cell numbers to ESAT-6 (*p* < 0.0001) (**A**) and CFP-10 (*p* < 0.0001) (**B**). The *P-value* was calculated using Mann Whitney test. ***: *p* < 0.001


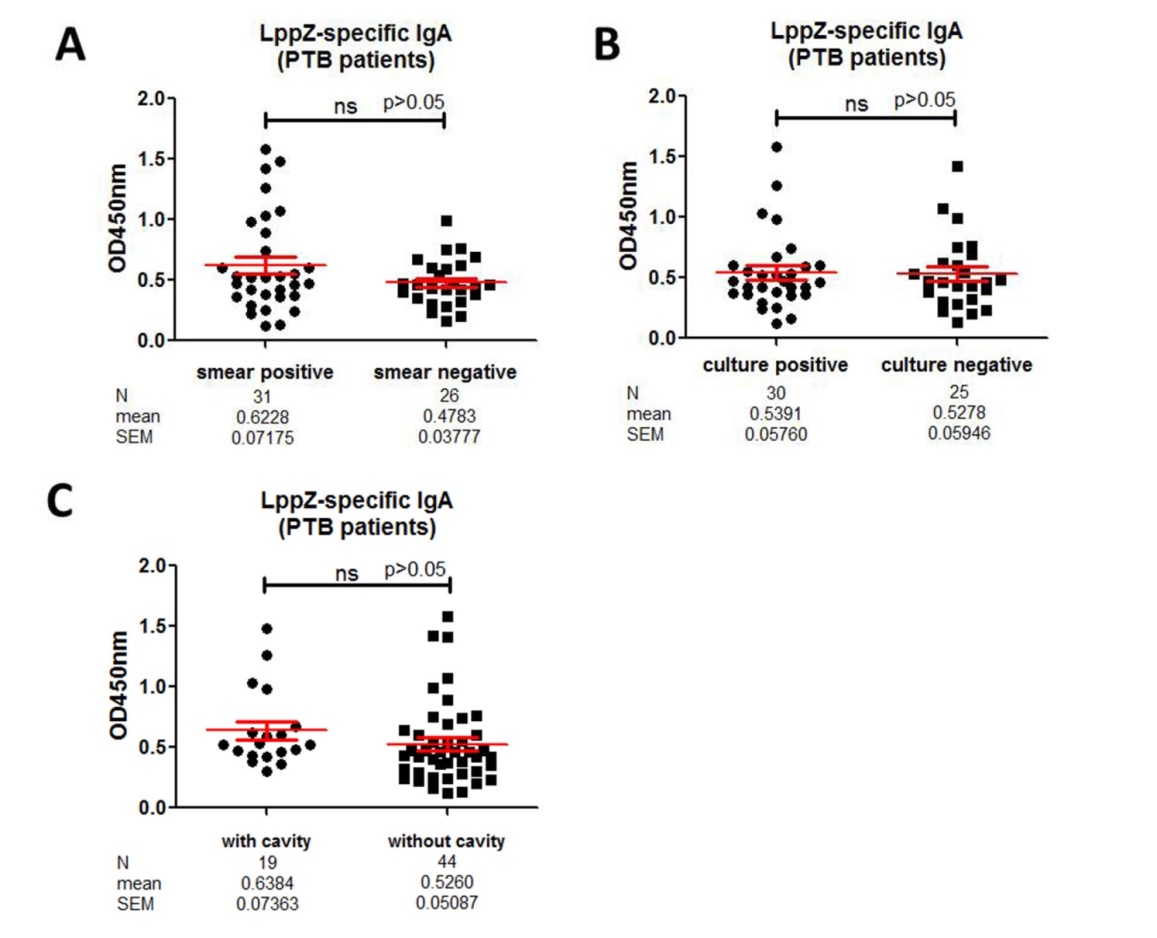


**Supplementary Figure 4 Analysis of LppZ-specific IgA levels with disease severity in PTB patients**

(**A**) Comparison of LppZ-specific IgA in the plasma between smear-positive PTB patients (N=31) and smear-negative PTB patients (N=26).

(**B**) Comparison of LppZ-specific IgA in the plasma between culture-positive PTB patients (N=30) and culture-negative PTB patients (N=25).

(**C**) Comparison of LppZ-specific IgA in the plasma between PTB patients with cavity (N=19) and PTB patients without cavity (N=44).

The *P-value* was calculated using Mann-Whitney test.
